# Nanomaterials for targeted drug delivery for immunotherapy of digestive tract tumors

**DOI:** 10.3389/fimmu.2025.1562766

**Published:** 2025-03-05

**Authors:** Mingzhu Li, Ningxin Li, Haozhe Piao, Shengbo Jin, Hongzhe Wei, Qian Liu, Jun Yu, Wenping Wang, Siyao Ma, Yuxin Jiang, Huini Yao, Yue Shen, Jiaqing Fu

**Affiliations:** ^1^ Cancer Hospital of China Medical University, Liaoning Cancer Hospital & Institute, Shenyang, Liaoning, China; ^2^ China Medical University, Shenyang, China; ^3^ Liaoning University of Traditional Chinese Medicine, Shenyang, China

**Keywords:** nanomaterials, immunotherapy, digestive tract tumors, drug delivery, application

## Abstract

The incidence and mortality rates of digestive tract tumors, especially gastric and colorectal cancers, are high worldwide. Owing to their unique advantages, such as efficient drug loading, safety, and targeting properties, nanoparticles (NPs) have demonstrated great potential in the treatment of gastrointestinal tumors. However, their practical application is limited by several factors, such as high costs, few clinical trials, and long approval periods. In this review, we summarize three types of immunotherapeutic nanomaterial drugs for gastrointestinal tumors: organic, inorganic, and hybrid nanomaterials. This article also discusses the current status of research and development in this field and the advantages of each type of material to provide theoretical references for developing new drugs and advancing clinical research.

## 1 Introduction

Gastrointestinal tract tumors originate in the digestive tract and include those resulting from esophageal, gastric, and colorectal cancers (1). In recent years, the incidence and mortality rates of gastrointestinal tumors have continued to increase. According to the International Agency for Research on Cancer (IARC), approximately 20 million new cancer cases were reported in 2022, including 1.926 million (9.6%) cases of colorectal cancer, 968,000 (4.9%) cases of gastric cancer, and 511,000 (2.6%) cases of esophageal cancer. Gastrointestinal tumors can grow into the lumen, obstructing the cavernous ducts and penetrating the wall, which may lead to obstruction, perforation, and even life-threatening complications (2, 3). Compared with traditional treatment modalities, such as surgery, radiotherapy, and chemotherapy, immunotherapy can yield more precise treatment effects by activating and promoting the ability of the immune system to recognize and kill tumor cells (4). However, immunotherapy remains subject to adverse reactions, such as autoimmunity or nonspecific immunity; moreover, its efficacy varies across patients. Therefore, developing advanced biomaterials for the delivery of immunotherapeutic drugs is necessary (5).

Nanoparticles (NPs), materials with a size of 1–100 nm, have been widely used in immunotherapy owing to their high surface-to-volume ratio, selectivity, sensitivity, and stability. Nanomedicines can be used to increase treatment efficacy, reduce toxicity, and enhance stability by delivering drugs to specific, targeted sites and stimulating immune cells and organs to produce sustained responses (6, 7). However, the use of nanoparticles for adjuvant immunotherapy has certain disadvantages, such as high costs, autotoxicity, and the delivery of drugs to nontarget cells (8). NPs can be strategically functionalized in accordance with the diverse characteristics of the digestive tract, including factors such as pH, the composition of the intestinal mucus layer, and the intestinal absorption capacity. This review summarizes the mechanisms of nanoparticles in immunotherapy and the applications and advantages of organic, inorganic, and hybrid nanoparticles in immunotherapy, providing important clinical and experimental references for developing novel nanoparticle-based drug delivery systems.

## 2 Advantages and mechanisms of action of nanomedicines

**Figure 1:**
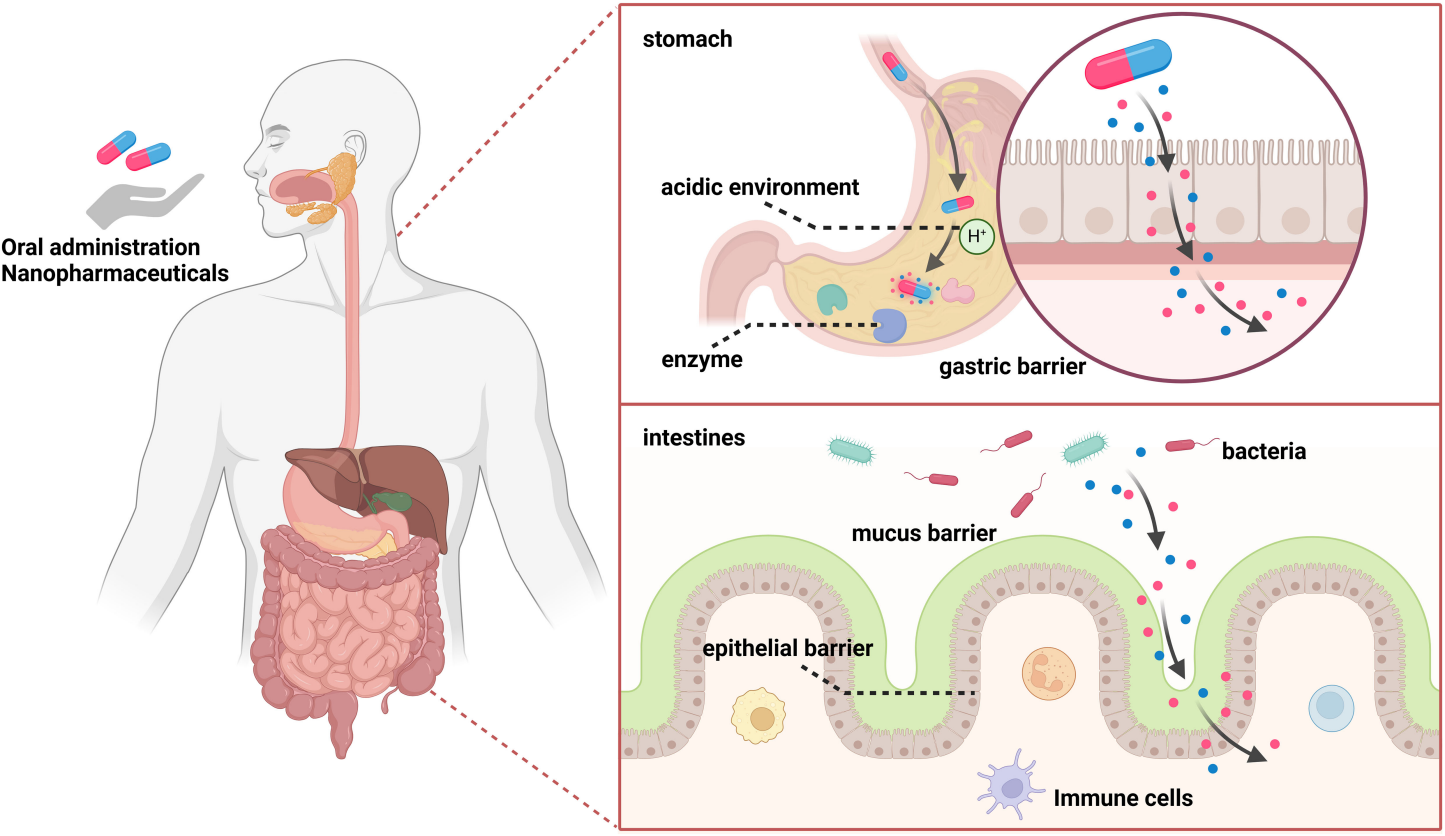
The gastrointestinal barrier mainly includes the gastric barrier, mucus barrier, and epithelial barrier. On one hand, a substantial pH gradient exists, ranging from 1-2.5 in the stomach to 7-8 in the colon. On the other hand, luminal enzymes and microorganisms in the stomach and duodenum can affect the stability of nanocarriers. Intestinal epithelial cells can target drugs to cross the epithelial barrier and deliver them into cells through transcellular or paracellular pathways.

The digestive tract constitutes the exclusive pathway through which orally administered nanomedicines are absorbed and subsequently enter systemic circulation via both the blood and lymphatic vessels. After successfully crossing the gastrointestinal barrier, which consists mainly of gastric, mucus and epithelial components, most nanocarriers enter the systemic circulation and undergo first-pass metabolism in the liver. On the one hand, a substantial pH gradient exists in the gastrointestinal tract, ranging from 1-2.5 in the stomach to 7-8 in the colon. On the other hand, enzymes in the lumen and microorganisms in the stomach and duodenum can affect the stability of nanocarriers. Additionally, intestinal epithelial cells can allow targeted drugs to cross the epithelial barrier for delivery into cells through transcellular or paracellular pathways (**Figure 1**) (9–11).

**Figure 2:**
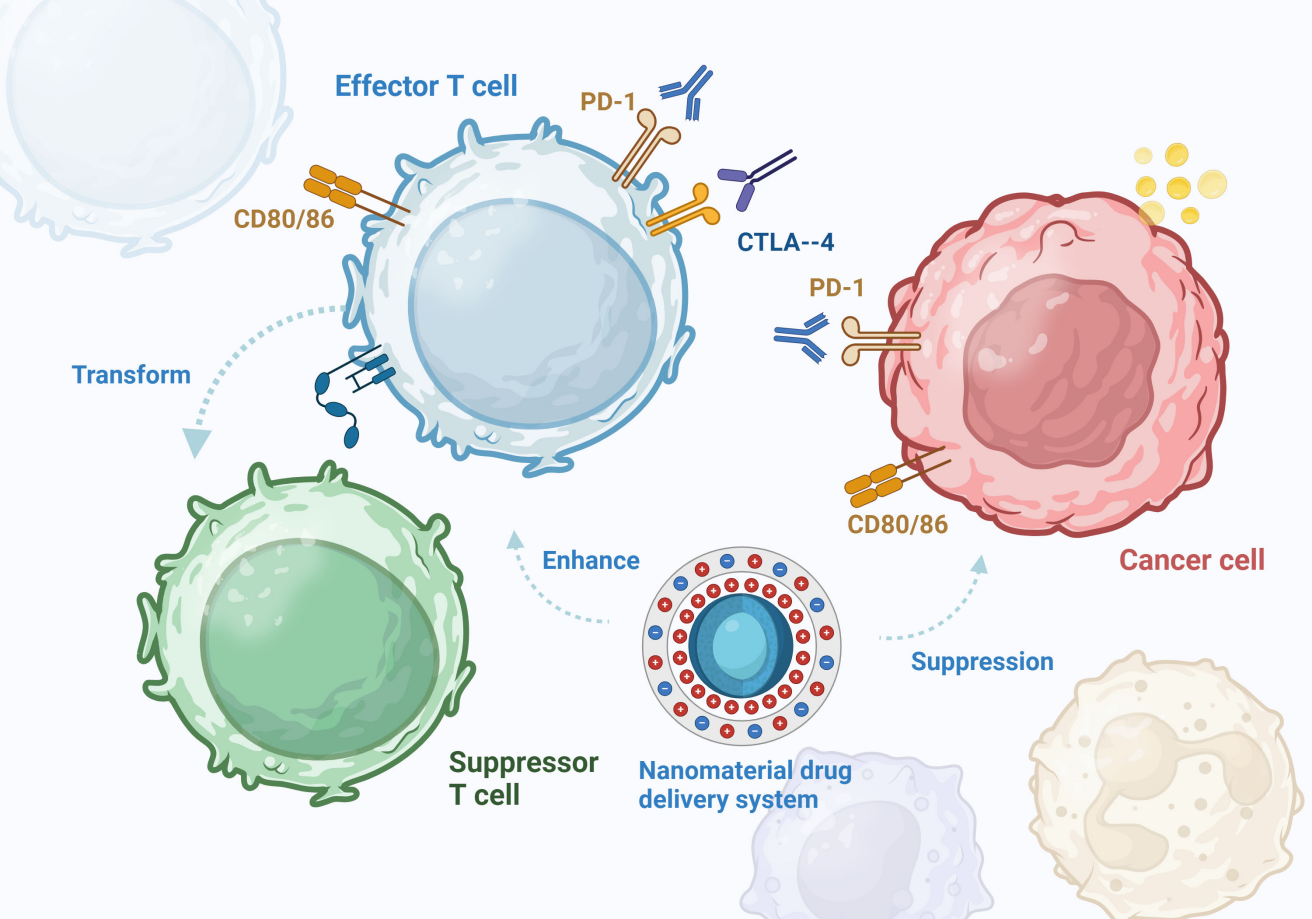
In the tumor microenvironment, PD-1 on the surface of T cells binds to PDL-1 on tumor cells, resulting in abnormally elevated PDL-1 expression. CTLA-4 binds to CD80 and CD86 to suppress antigen-presenting cells. The effector T cells are converted to suppressor T cells under tumor action, reducing the body’s anti-tumor immune response. Nanomaterials can assist immunotherapy, enhance immunotherapy effect and induce immunogenic death of tumor cells.

The tumor microenvironment (TME) plays important roles in tumor development, progression, and treatment and serves as a crucial site of tumor cell metabolism. NP-mediated drug delivery systems have been applied to target various types of immune cells. The TME contains macrophages, neutrophils, myeloid suppressor cells, dendritic cells, natural killer cells, T and B cells, and several noncellular components (12). The application of a nanomedicine delivery system with active targeting capabilities could further enhance the immunostimulatory capabilities of the drug in the TME (13) (**Figure 2**). NPs are smaller than 100 nm, which facilitates their dispersion in the body and diffusion into the site of action through the blood circulation or the lymphatic system. Upon reaching the target cell, NPs recognize specific receptors on the cell surface, which allows their entry into the cell. Inside the cell, NPs rely on cellular structures and functions, such as vesicular transport and direct diffusion, to reach the target site for drug release (14). NPs can effectively deliver drugs to immune cells and increase their immunogenicity to achieve the desired effects of adjuvant immunotherapy. NPs can regulate the proportions of various immune cells in the tumor immune microenvironment and induce immunogenic tumor cell death (15). NPs can simultaneously carry various immunomodulators and continuously release them into tumor tissues and lymph nodes, effectively activating antitumor immunity and influencing tumor antigen production, APC activation, and immune checkpoint inhibition (16). In addition, NPs can be used to induce iron-dependent cell death and apoptosis, as different tumors have different sensitivities to iron. Taken together, these data indicate that NPs can increase the efficacy of immunotherapy by targeting various cellular processes (17).

## 3 Nanomaterial applications

**Figure 3:**
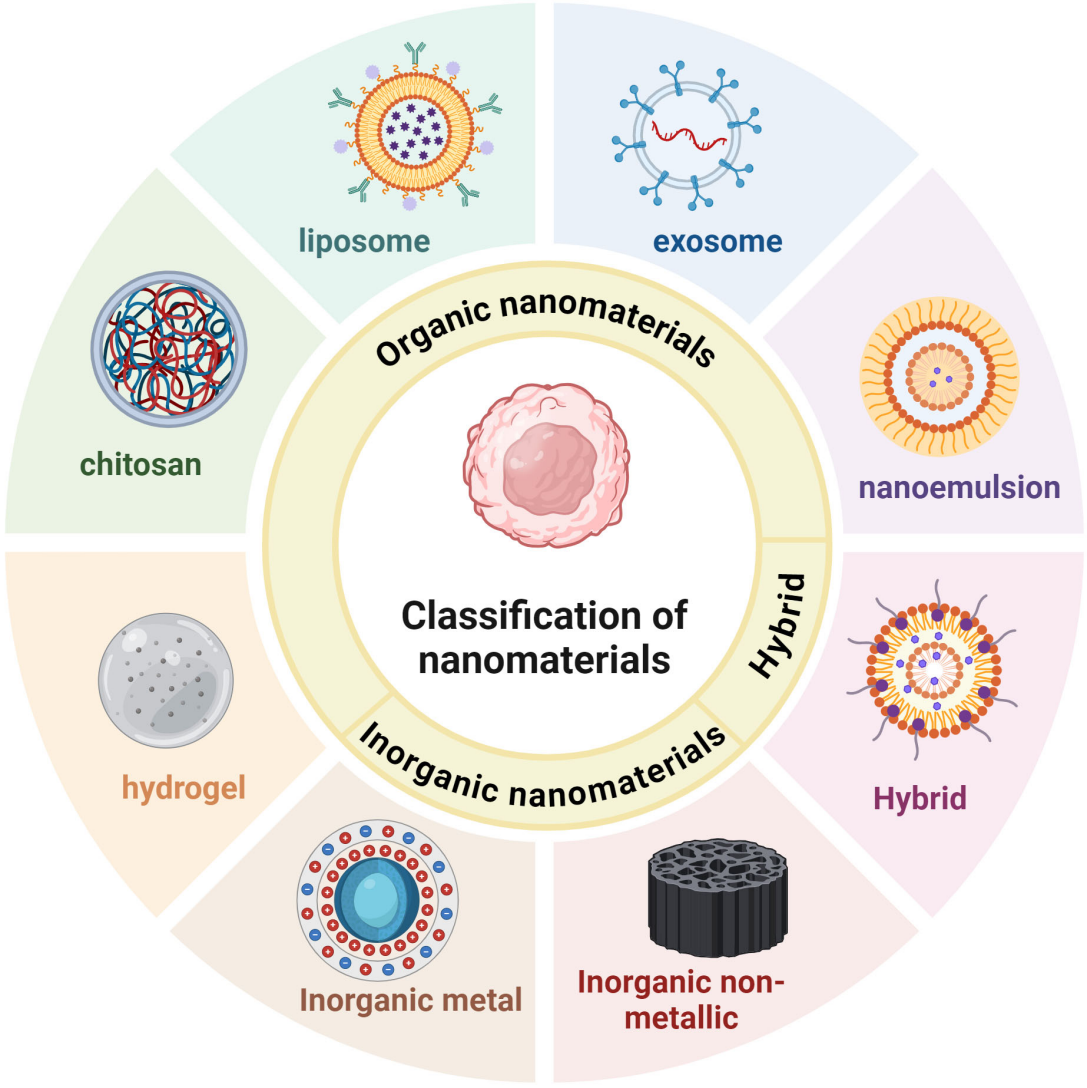
Nanomaterials used for gastrointestinal tumors immunotherapy include organic nanomaterials, inorganic nanomaterials, and hybrid nanomaterials. Organic nanomaterials mainly include hydrogels, chitosan, liposomes, exosomes, and nanoemulsions. Inorganic nanomaterials can be further categorized into inorganic metallic nanomaterials and inorganic non-metallic nanomaterials. Inorganic metallic nanomaterials such as gold-based, silver-based, copper-based, etc.; inorganic non-metallic nanomaterials such as silica, selenium, graphene, etc.

Several oral antitumor drugs are severely affected by digestion in the gastrointestinal tract. The encapsulation of antitumor drugs in NPs can effectively prevent premature drug release in the upper gastrointestinal tract but allow the drug to be released once the target tumor cell or site is reached (18). Carrier-based nanomedicines include active pharmacological ingredients (APIs), carrier materials, and other inactive ingredients. Selecting an appropriate nanomaterial on the basis of the site of drug action, pathways involved, and pH can effectively improve drug release. Organic nanomaterials have high biocompatibility, good targeting ability, low toxicity, and the ability to load multiple drugs (19) (**Table 1**). The digestive tract, including the esophagus, stomach, and intestines, is lined with a mucous membrane. Owing to their physical properties, organic nanomaterials can adhere to these membranes, thus enhancing the penetration and absorption of their encapsulated drugs (20). Inorganic nanomaterials have unique electrical, optical, magnetic, and thermal properties; high absorption and stability; and a long duration of retention in the host (21) (**Table 2**). The following sections discuss the advantages and current research status of organic (hydrogels, chitosan, liposomes, exosomes, and nanoemulsions), inorganic (metallic, nonmetallic, gold, graphene, and phosphorus), and hybrid nanomaterials in immunotherapy (**Figure 3**).

**Table 1:** Organic nanomaterials for cancer treatment.

Organic nanomaterial	Type of cancer treated	Advantages	Reference(s)
hydrogels	colorectal cancer gastric cancer	1. Low cytotoxicity to normal cells and produces significant and sustained inhibitory effects on cancer cells. 2. Inhibits reactive oxygen species and the generation of singlet oxygen radicals in tumor cells. 3. Improves drug uptake by cells. 4. Good stability, temperature sensitivity, and carrier performance.	(25–28)
chitosan	colorectal cancer gastric cancer	1. Bioadhesive properties and high permeability. 2. Reduces reactive oxygen species production and inhibits of tumor cell growth. 3. Modifies the characteristics of chemotherapy drugs, including half-life, toxicity, circulation time, etc. 4. Improves cellular uptake, prolongs drug release duration, and increases drug accumulation.	(32, 33)
liposomes	colorectal cancer Cholangiocarcinoma gastric cancer	1. Reduces immunosuppressive cytokine levels and induces tumor-specific antigen presentation. 2. Regulates hypoxic TME, alleviates hypoxia-related immunosuppression and enhances the antitumor immune response. 3. Enhances mitochondria-associated iron death, increases phagocytosis by dendritic cells and macrophages and induces cross-presentation of antigens. 4. Accumulates in tumor cells and initiates CD8+ T-cell infiltration into the TME. 5. Induces immunogenic tumor cell death and retards tryptophan metabolism.	(37–40)
exosomes	gastric cancer	1. Strong penetration ability, long blood circulation time, and good biocompatibility. 2. Activates CD4+ or CD8+ T cells, inducing cells to produce an adaptive immune response.	(44)
nanoemulsions	colorectal cancer gastric cancer	1. High selectivity, low toxicity, significantly improves drug efficacy and shortens drug delivery time. 2. Enhances the body’s immune response to tumors through the immunomodulatory cell signaling pathway.	(47, 48)

**Table 2:** Inorganic nanomaterials for cancer treatment.

Inorganic nanomaterial		Type of cancer treated	Advantages	Reference(s)
metal	metals	gastric cancer	1. Good stability, narrow particle size distribution, adjustable morphology. 2. Enables the drug to produce direct toxic effect on tumor cells.	(50)
	copper	colorectal cancer	1. Increases ROS levels in tumor cells and induces oxidative stress. 2. Induces apoptosis in tumor cells.	(51)
	silver	colorectal cancer	1. Better biocompatibility than other metal-based nanoparticles. 2. Higher toxicity to tumor cells, leading to a significant increase in intracellular ROS levels.	(53)
	calcium	colorectal cancer	1. Initiates cellular pyroptosis and remodeling of the immunosuppressive TME. 2. Synergistically polarizes tumor-associated macrophages toward the M1 phenotype and attenuates the immunosuppressive TME.	(54)
Nonmetallic	silica	gastric cancer colorectal cancer	1. Uniform pore size, controllable particle size, large surface area, and high stability. 2. Selective killing of tumor cells with low toxicity to normal cells. 3. Releases the drug only after endocytosis by tumor cells and reaching the lysosome, reducing drug waste. 4. Reduces adverse drug reactions.	(56, 57)
	selenium	gastric cancer	1. Induces the production of ROS, causing oxidative stress. 2. Higher toxicity to tumor cells and no toxicity to normal cells. 3. Induces autophagy and apoptosis by modulating signaling pathways.	(60)
	Graphene	gastric cancer	1. Large surface area, flexible structure, easy modification, good biocompatibility and low toxicity. 2. Reduces sudden drug release.	(62)

### 3.1 Organic nanomaterials

#### 3.1.1 Hydrogels

Owing to their inherent properties that effectively control drug release, hydrogels are frequently formulated as extremely small particles at the nanometer scale, known as hydrogel nanoparticles. These nanoparticles consist of polymer molecules, fibers, or particles arranged in a 3D mesh structure, which encapsulates drugs within its internal network (22). By integrating the benefits of its constituent materials, these nanoparticles not only preserve the integrity and functionality of the resulting nanomaterial but also enhance the drug release capabilities and modifiability (23, 24). Yuan et al. used silk fibroin (SF) encapsulating curcumin (CUR) and 5-fluorouracil (5-FU) to synthesize highly efficient drug-carrying hydrogels to fabricate composite drug-carrying nanospheres with particle sizes ranging from 77.87 nm to 299.22 nm. The CUR hydrogel exhibited low cytotoxicity in a normal human colon epithelial cell line (NCM) but exerted a significant and sustained inhibitory effect on the growth of a human colon cancer cell line (HT-29). These results indicate that SF-based drug-loaded composite hydrogels have advantages in the treatment of colorectal cancer (25). Similarly, Zhang et al. encapsulated CUR in NPs and embedded these NPs in GelMA/SilMA hydrogels for the treatment of colorectal cancer. These composite hydrogels improved the cellular uptake of CUR. Shanmugapriya synthesized a fucoidan-based hydrogel (FAG@H) by mixing fucoidan, alginate, and a hydrogel in a 3:3:1 ratio. FAG@H inhibits reactive oxygen species and generates single linear oxygen radicals in gastrointestinal tumor cells. Both of these hydrogels represent a more efficient drug delivery system by improving stability and bioavailability (26, 27). A majority of hydrophobic drugs cannot be successfully transported to the colon. To overcome this challenge, Abbasi et al. synthesized folic acid-conjugated nanoparticles (FNPs) and incorporated them into a pH-sensitive hydrogel via free radical polymerization. After oral administration, this composite hydrogel efficiently delivered hydrophobic drugs to colorectal tumor cells (28).

#### 3.1.2 Chitosan

Chitosan, also known as deacetyl chitin, consists of β-1,4-D-glucosamine and N-acetyl-D-glucosamine units. It is nontoxic or minimally toxic, has good bioactivity and degradability, and is considered safe and biocompatible (29). Chitosan adheres to the mucosa due to the formation of electrostatic interactions between its positively charged amino groups and the negatively charged salivary acid in the mucosal membrane of the digestive tract. These interactions effectively prolonged the retention of chitosan in colonic epithelial cells (30). Chitosan-based materials can not only deliver antitumor drugs to the target site but also enhance the cytotoxicity of these drugs. Studies have shown that chitosan NPs can alter certain properties of chemotherapeutic drugs, such as their half-life, toxicity, circulation time, and release profile (31). Bhirud et al. prepared chitosan-based nanoparticles coated with tocopheryl polyethylene glycol 1000 succinate (TPGS). The adhesive properties of chitosan and the high permeability of TPGS allowed the efficient delivery of imatinib mesylate (IMT) to colon cancer cells. The IMT-loaded chitosan NPs coated with TPGS (CS-IMT-TPGS-NPs) were effectively internalized by cells, released IMT in a controlled and sustained manner, and increased the accumulation of IMT in cancer tissues. In addition, CS-IMT-TPGS-NPs suppressed the production of reactive oxygen species, thereby inhibiting colon cancer cell growth (32). Small molecule alkaloids exhibit antitumor activity, inhibit tumor cell proliferation and induce tumor cell apoptosis. However, they have low bioavailability and poor solubility. Babaeenezhad encapsulated small molecule alkaloids within chitosan NPs, which enabled their delivery to gastric cancer cells and improved the anticancer properties of the drug itself (33).

#### 3.1.3 Liposomes

**Figure 4:**
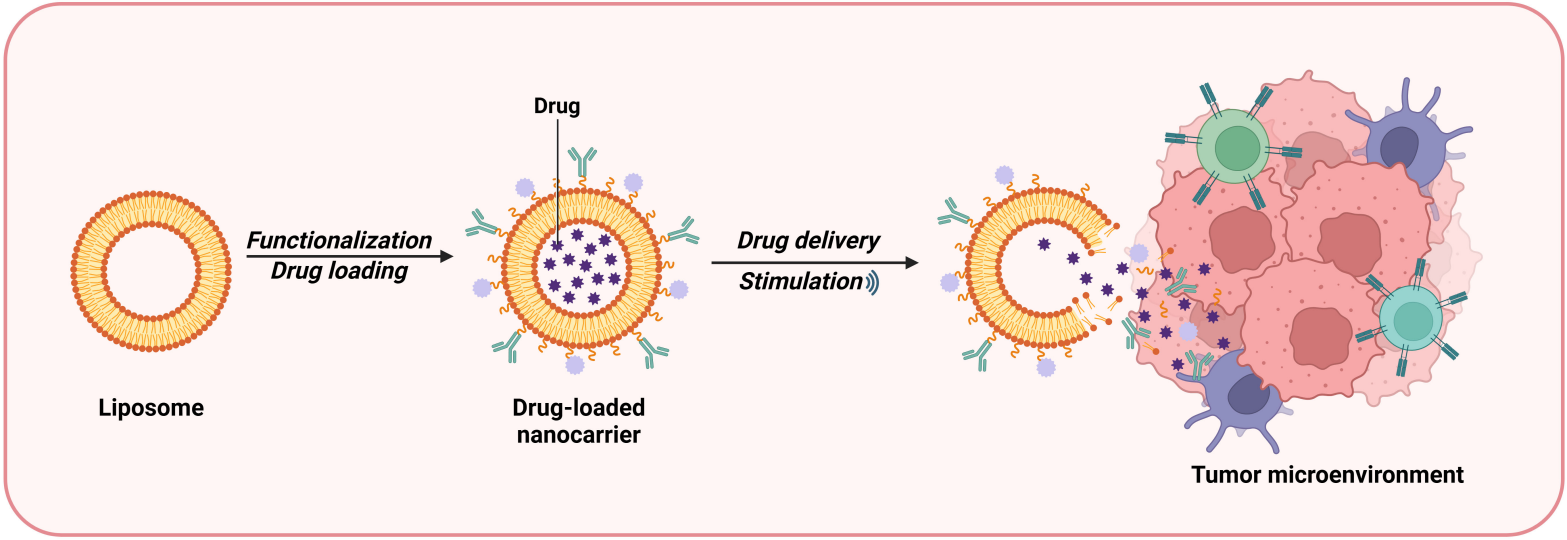
Liposomes are a type of bilayer lipid vesicle liposome prepared from lipid materials that can be used as drug carriers, with a hydrophilic environment and phospholipid molecular layer structure inside. The nanosystem of liposomes can target and inhibit TME. It can promote the uniform infiltration of NP into TME by targeting innate immune suppressive immune cells and stromal cells.

Liposomes are spherical vesicles composed of lipid bilayers with good biocompatibility and safety that can be used as drug carriers. Owing to their internal hydrophilic environment and phospholipid bilayer structure, liposomes can efficiently deliver both hydrophilic and lipophilic drugs (34). Lipid nanocarriers can bypass the first-pass metabolism of the liver and enter the systemic circulation directly via the intestinal lymph nodes, thereby improving the bioavailability of the encapsulated material (35). Liposome-based nanodrug delivery systems can be used to inhibit the TME in a targeted manner. Liposome-based systems can allow the uniform entry of NPs into the TME by targeting immunosuppressive innate immune cells and stromal cells (**Figure 4**). These systems can also inhibit immunosuppressive cytokines and induce tumor-specific antigen presentation, increasing the sensitivity of tumor cells to attacks by immune cells. In addition, Liposome-based drug delivery systems can deliver molecules such as hemoglobin to attenuate hypoxia-associated immunosuppression, enhancing antitumor immune responses (36). Shen et al. constructed bifunctional liposomes encapsulating an oxaliplatin prodrug and an indoleamine-2,3-dioxygenase (IDO) inhibitor. These liposomes not only induced immunogenic death in cancer cells but also inhibited tryptophan metabolism, reshaping the immune microenvironment of colorectal cancer, increasing the number of CD8+ T cells and enhancing the cytotoxicity of the drugs, resulting in better therapeutic effects against colorectal cancer (37, 38). Jiang et al. used liposomes to codeliver etoposide (ETP) and CUR for the treatment of gastric cancer. Their results showed that the two drugs exerted synergistic effects upon release from the liposomal carrier system. The drugs exhibited high cytotoxicity and enhanced antitumor activity *in vitro* and *in vivo* and low toxicity to normal tissues (39). A randomized controlled trial performed in Germany was conducted to evaluate the efficacy of liposomal irinotecan for the treatment of biliary tract cancer. A total of 91 patients with advanced cholangiocarcinoma were randomly divided into two groups: the nanoliposomal irinotecan/fluorouracil/folinic acid (experimental) group and the conventional first-line treatment regimen (control) group. The results revealed that the progression-free survival (PFS) of the experimental group was 51%, with an objective response rate of 24.5%, whereas the objective response rate of the control group was 11.9%. These findings suggest that liposomes hold promise in the treatment of advanced cholangiocarcinoma (40).

#### 3.1.4 Exosomes

Exosomes are nanosized lipid bilayer-containing vesicles secreted by cells. They contain various biological components, such as proteins, lipids, and nucleic acids, in their lumen or lipid bilayer (41). The structures and properties of exosome-based drug delivery systems are similar to those of liposomes. Exosomes can carry both hydrophilic and hydrophobic drugs and have unique advantages, such as strong penetration ability, a long blood circulation time, and good biocompatibility (42).

Exosomes produced by dendritic cells (DCs) and macrophages are anti-immunogenic. Exosomes produced by dendritic cells can activate CD4+ or CD8+ T cells via the peptide–major histocompatibility complex (MHC), thereby inducing adaptive immune responses in these cells (43). Li et al. fused induced pluripotent stem cell (iPSC)-derived exosomes with DC-derived exosomes for the diagnosis and treatment of gastric cancer. The presence of a modified anti-PD-1 (aPD-1) antibody induced the release of suppressed T lymphocytes. In addition, the fused exosomes recruited immune cells to kill tumor cells and exposed many tumor-associated antigens, effectively enhancing the efficacy of immunotherapy (44).

#### 3.1.5 Nanoemulsions

Nanoemulsions are stable, transparent, low-viscosity dispersion systems with particle sizes <100 nm. They are composed of an aqueous phase, an oil phase, emulsifiers, and coemulsifiers at a certain ratio (45). Nanoemulsions can carry both hydrophilic and hydrophobic compounds and improve the solubility and stability of the encapsulated drugs, prolong their retention, increase their bioavailability, and deliver them in a targeted manner (46). Tong et al. developed a DC vaccine comprising a tubeimoside I (TBI)-encapsulated nanoemulsion for treating colorectal cancer. The results revealed that the nanoemulsion exhibited high selectivity and low toxicity, significantly improved the efficacy of TBI, and shortened the drug administration time (47). Astaxanthin-based nanoemulsions composed of hydrophilic active agents, bioactive complexes, and emulsifiers have been used to treat gastric and colorectal cancers. Low levels of free radicals have been shown to have therapeutic effects on related diseases. The aforementioned nanoemulsions showed high radical scavenging capacity at low concentrations and enhanced antitumor immune responses by modulating immunomodulatory signaling pathways, effectively inhibiting tumor cell growth (48).

### 3.2 Inorganic nanomaterials

#### 3.2.1 Metallic nanomaterials

Gold NPs have good biocompatibility and low toxicity. They are widely used as carriers for delivering drugs and diagnosing tumors owing to their advantages of good stability, small particle size, and modifiable morphological features (49). Antibodies can improve the recognition, uptake, and accumulation of nanodrug delivery systems both *in vitro* and *in vivo*. Fan reported that gold NPs encapsulating ravulizumab exerted synergistic toxic effects to gastric cancer cells but did not cause damage to normal gastric cells. In addition, the expression of high-affinity immunoglobulin γ Fc receptor I was greater in gastric cancer cells treated with ravulizumab-encapsulated gold NPs than in cells treated with ravulizumab alone (50). Ghasemi reported that copper-based NPs increased ROS production and induced oxidative stress in the human colorectal cancer cell line SW480. Furthermore, these copper-based NPs induced apoptosis in SW480 cells by targeting the mitochondrial pathway, decreasing the protein expression of Bcl-2, and increasing the protein expression of Bax and p53 (51). Silver-based NPs have demonstrated better biocompatibility than other metal-based NPs (52). Taati evaluated the effects of silver-based NPs encapsulating glutamine and thiosemicarbazone on colon cancer cells. The results showed that these NPs had greater cytotoxicity and led to a significant increase in ROS levels in SW480 cells. Mechanistically, these silver-based NPs effectively inhibited the SW480 cell growth by damaging DNA and other important biomolecules and disrupting the cell cycle (53).

Pyroptosis, a programmed cell death mode that occurs in macrophages, is a novel target for inducing antitumor immunity. Calcium-based, intelligent-responsive nanoinducers (CaZCH NPs) were used to induce pyroptosis in cells and remodel the immunosuppressive TME to increase the efficacy of colorectal cancer immunotherapy. CaZCH NPs are degraded in the acidic TME, leading to the rapid release of Ca^2+^, CUR, H_2_O_2_ and O_2_. These degradation products lead to mitochondrial damage and promote H_2_O_2_-induced oxidative stress. The O_2_ produced by CaZCH NPs and the proinflammatory cytokines released by pyroptotic cells synergistically polarize tumor-associated macrophages toward the M1 phenotype, thereby attenuating immunosuppression (54).

#### 3.2.2 Nonmetallic nanomaterials

Silica NPs are characterized by a uniform pore size, controllable particle size, and large surface area. Compared with conventional drug delivery systems, silica NPs have greater stability when exposed to fluctuating temperatures, in the presence of organic solvents, and under acidic conditions (55). Wang et al. used polydopamine-encapsulated mesoporous silica NPs to deliver gefitinib to gastric cancer cells. These NPs selectively killed gastric cancer cells with minimal toxic effects on normal gastric cells. The NPs adhered to the gastric mucosa, enabled the sustained release of gefitinib, and inhibited burst release of the drug (56). A silica-based drug delivery system was used to treat colorectal cancer. This system exhibited enhanced anticancer effects through CD44 receptor-mediated endocytosis and significantly inhibited tumor growth (57). Compared with traditional selenium compounds, selenium nanoparticles have stronger biological activity and lower toxicity and can regulate different immune cell functions (58). In addition to serving as anticancer drug carriers, selenium NPs can induce ROS production and oxidative stress in tumor cells (59). Wang et al. reported that black ginger–selenium NPs are toxic to human gastric adenocarcinoma cells but do not have significant toxic effects on normal gastric cells. Mechanistically, selenium NPs induce autophagy and apoptosis in gastric adenocarcinoma cells through the phosphatidylinositol 3-kinase (PI3K) signaling pathway, thereby exerting antitumor effects (60). Graphene is a novel two-dimensional carbon-based nanomaterial characterized by a large surface area, flexible structure, ease of modification, good biocompatibility, and low toxicity. Graphene can be transported to the tumor site through the blood circulation, resulting in its accumulation in tumor tissues through passive targeting (61). Yaghoubi evaluated the effects of graphene oxide carrying CUR and adriamycin on human gastric cancer cells. Research confirmed that graphene oxide has better biocompatibility in low-concentration environments. After treatment with graphene oxide, the sudden release of drugs is reduced, biocompatibility is enhanced, and the toxic side effects of drugs to normal cells are effectively decreased (62).

### 3.3 Hybrid nanomaterials

Hybrid nanomaterials contain both organic and inorganic NPs, combining the advantages of both materials to maximize the effects of the loaded antitumor drugs. Hou synthesized a nanocomposite hydrogel using graphene oxide and polyvinyl alcohol to deliver drugs to colon cancer cells. Polyvinyl alcohol is a nontoxic and biocompatible hydrophilic polymer; however, the amount of polyvinyl alcohol loaded into the hydrogels was limited, and polyvinyl alcohol was not uniformly distributed. Moreover, the macroporous structure of hydrogels causes rapid drug release. Given that graphene forms tight aggregates in acidic environments to limit drug release and that the colon and rectum have neutral pH values, Hou used graphene as an ideal complement to the polyvinyl alcohol-encapsulated in hydrogels. The nanocomposite hydrogel containing graphene oxide and polyvinyl alcohol efficiently passed through the stomach and small intestine and reached the colon intact. The hydrogel exhibited significantly improved therapeutic effects and provided an efficient and low toxicity drug delivery system for the treatment of colorectal cancer (63).

## 4 Discussion

Due to rapid advancements, tumor treatment modalities are becoming more diverse. Although traditional treatment strategies such as surgery, radiotherapy, and chemotherapy have certain therapeutic effects, they also have several limitations that should be addressed. The clinical application of chemotherapy is limited owing to the high risk of adverse reactions and low selectivity. Targeted therapy and immunotherapy have recently been developed as treatment strategies for cancer. Immunotherapy involves the use of the patient’s immune system to recognize and kill tumor cells. Although ICIs have made substantial progress in the treatment of various cancers, their adverse effects, such as large differences in efficacy among patients, drug resistance, and frequent disease recurrence, pose challenges to their successful clinical application (64). Owing to their controllable size, high biocompatibility, strong targeting ability, and minimal adverse effects, nanomaterials have been widely used to address the limitations of other cancer treatment strategies, such as chemotherapy, immunotherapy, gene therapy, and photothermal therapy (65). Numerous *in vitro* and *in vivo* studies have shown that nanomaterials can increase the efficacy of PD-1 inhibitors by alleviating hypoxia in the TME, inducing systemic antitumor immune responses, and modulating the TME (66). In addition, some nanomaterials can influence the metabolism of the intestinal flora to reduce inflammation and promote intestinal barrier repair. Nanomaterials can indirectly exert antitumor effects by modulating the function of the intestinal flora, which is a novel strategy for the treatment of digestive tract tumors (67).

The optimal nanomaterial or combination of nanomaterials should be selected on the basis of the pathogenesis of the particular tumor to be treated and the advantages of each type of nanomaterial to prevent the early release of drugs under physiological conditions. The pH of the stomach usually ranges from 0.9 to 1.8; therefore, nanomaterials that are sensitive to and efficiently decompose in an acidic environment should be used as drug carriers in the treatment of gastric cancer. For example, the amino group of chitosan is protonated in a low pH environment and increases the osmotic pressure, which is more favorable for acid-sensitive drug release (68). In contrast, the pH of the colon and rectum is neutral. Nanocarriers and encapsulated drugs are required to pass through the acidic environment of the stomach to reach the colon or the rectum. Therefore, nanomaterials that are less susceptible to acidic environments and can release drugs in neutral environments should be used as drug carriers in the treatment of colorectal cancer. For example, graphene oxide-based composite nanomaterials can efficiently protect the loaded drug while passing through the stomach and small intestine to reach the proximal colon. These nanomaterials have enhanced colon-targeting abilities and prolonged retention at the target site (63). Organic nanomaterials can be used for precision therapy by selecting appropriate nanomaterials for different types of tumors on the basis of their pathogenesis and characteristics.

Although nanotechnology has made remarkable progress, its practical application is limited by several factors. When drug-encapsulated NPs enter the immune system, they affect the ability of DCs to induce the differentiation of T cells into T helper 17 (Th17) cells, generating an immune response. The immune system recognizes these NPs as foreign substances and generates an immune response against them (69). The therapeutic index should be considered when designing nanomaterials to prevent the adverse effects of hypersensitivity reactions in patients. How to prepare a stable and nontoxic drug delivery system, how to precisely control drug release, and how to evaluate transport efficiency *in vivo* are unaddressed concerns. To address the limitations of traditional drug delivery systems, such as low solubility and poor safety and efficacy, nanodrug delivery systems, such as the representative liposomal adriamycin, are emerging for the treatment of many types of tumors. However, developing these systems is expensive, and their clinical translation relies on evidence from clinical trials and a lengthy approval process (70). Therefore, reducing the production costs and ensuring therapeutic efficacy are key and must be addressed in the future. The development of nanomaterials requires the integration of multiple disciplines, including medicine, biology, chemistry, and materials science. Therefore, strengthening interdisciplinary cooperation is essential for promoting the development of nanodrug delivery systems. In addition, nanomaterials should be designed to solve clinical problems instead of focusing on the multifunctionality of the delivery system (71). To date, most nanodrug delivery systems for cancer treatment have been evaluated in preclinical models, and only a few clinical trials have been conducted. Therefore, more clinical studies are warranted to promote the development and clinical application of novel nanodrug delivery systems for the treatment of cancer.
